# Non-canonical Fzd7 signaling contributes to breast cancer mesenchymal-like stemness involving Col6a1

**DOI:** 10.1186/s12964-020-00646-2

**Published:** 2020-09-07

**Authors:** Ping Yin, Yu Bai, Zhuo Wang, Yu Sun, Jian Gao, Lei Na, Zhongbo Zhang, Wei Wang, Chenghai Zhao

**Affiliations:** 1 Department of Pathophysiology, College of Basic Medical Science, China Medical University, Shenyang, China; 2 Department of Nephrology, Shengjing Hospital, China Medical University, Shenyang, China

**Keywords:** Frizzled 7, Stemness, Breast cancer, Lgr5, Wnt

## Abstract

Mesenchymal-like stemness is characterized by epithelial-mesenchymal transition (EMT). Breast cancer (BC) cell mesenchymal-like stemness is responsible for distal lung metastasis. Interrogation of databases showed that Fzd7 was closely associated with a panel of mesenchymal-related genes and a panel of stemness-related genes. Fzd7 knockdown in mesenchymal-like MDA-MB-231 and Hs578T cells reduced expression of Vimentin, Slug and Zeb1, induced an epithelial-like morphology, inhibited cell motility, impaired mammosphere formation and decreased Lgr5^+^ subpopulation. In contrast, Fzd7 overexpression in MCF7 cells resulted in opposite changes. Fzd7 knockdown delayed xenograft tumor formation, suppressed tumor growth, and impaired lung metastasis. Mechanistically, Fzd7 combined with Wnt5a/b and modulated expression of phosphorylated Stat3 (p-STAT3), Smad3 and Yes-associated protein 1 (Yap1). Moreover, Fzd7-Wnt5b modulated expression of collagen, type VI, alpha 1 (Col6a1). Both Wnt5b knockdown and Col6a1 knockdown disrupted BC cell mesenchymal phenotype and stemness. Taken together, Fzd7 contributes to BC cell EMT and stemness, inducing tumorigenesis and metastasis, mainly through a non-canonical Wnt5b pathway. Col6a1 is implicated in Fzd7-Wnt5b signaling, and mediates Fzd7-Wnt5b -induced mesenchymal-like stemness.

**Video Abstract**

**Video Abstract**

## Introduction

Frizzled (Fzd) are seven-transmembrane proteins harboring a conserved 120-amino-acid cysteine-rich domain (CRD), via which Fzd combine with extracellular Wnt molecules. Wnt/Fzd pair initiates β-catenin -dependent or independent signaling pathways. Wnt/β-catenin is one of the most important oncogenic pathways involved in many aspects of tumors such as proliferation, epithelial-mesenchymal transition (EMT), angiogenesis, chemoresistance, stemness and metastasis. Signaling initiated by Wnt molecules such as Wnt5a and Wnt5b, is independent on β-catenin, and conventionally called non-canonical Wnt pathway. Actually, non-canonical Wnt pathway is also related to EMT, stemness and metastasis [1–3].

There are 10 members identified in human Fzd family. Fzd7 is implicated in human cancers. Fzd7 was upregulated in hepatocellular carcinoma (HCC), resulting in activation of β-catenin pathway, accompanied by increased TCF transcriptional activity and cell proliferation rate [4–6]. Fzd7/β-catenin pathway was also activated in triple-negative breast cancer (TNBC), responsible for cell proliferation [7, 8]. However, recent studies demonstrated that Fzd7 also mediated non-canonical Wnt signaling. Wnt5a-Fzd7 pair promoted growth and tumorigenesis of melanoma [9]. Moreover, Fzd7 signaling induced gastric tumorigenesis irrespective of APC mutation status, suggesting this process might not be related to β-catenin [10].

Cancer cell stemness is related to distal metastasis, relapse and chemoresistance. Mesenchymal-like stemness is characterized by EMT [11]. EMT has been shown to induce mammary stem cell and tumor-initiating cell stemness [12, 13]. Oncogenic pathways such as IL-6/Stat3, EGFR, NOTCH and TGF-β1 are involved in breast cancer (BC) cell mesenchymal phenotype and stemness [14–17]. Interrogation of databases indicated that Fzd7-mediated non-canonical Wnt pathway cross-talked with these pathways, thereby suggesting Fzd7 might contribute to BC cell mesenchymal-like stemness. Interrogation of databases further identified Wnt5b as a potential ligand for Fzd7, and collagen, type VI, alpha 1 (Col6a1) as a downstream molecule in Fzd7-Wnt5b signaling.

## Methods

### In silico analysis

Cancer Cell Line Encyclopedia (CCLE) database and GSE12777 database were interrogated for gene expression in human BC cell lines. GSE2603 database was interrogated for gene expression in human BC tissues. Correlation between two genes was analyzed by Pearson statistics. Heat maps were generated by GraphPad Prism 7.0.

### Cell culture

MDA-MB-231, Hs578T, MCF7 and BT-549 cells were cultured in DMEM with 10% fetal bovine serum (FBS) and 1% penicillin/streptomycin at 37 °C and 5% CO2 in a humidified incubator. T47D cells were cultured in RPMI1640 with 10% fetal bovine serum (FBS) and 1% penicillin/streptomycin. All cell lines were obtained from ATCC.

### Western blot

Cells were washed twice with cold PBS and were lysed in RIPA buffer with Phenylmethanesulfonyl fluoride (both Beyotime). BCA Protein Assay Kit (Beyotime) was used to quantify protein concentrations. Proteins were separated by SDS-PAGE and transferred onto PVDF membranes (Immobilon-P). 5% bovine serum albumin was performed to block membranes. Primary antibodies include Fzd7 (Santa Cruz Biotechnology, sc-293,261), Col6a1 (Santa Cruz Biotechnology, sc-377,143), Wnt5a/b (Cell Signaling Technology, #2530), IL-6 (Cell Signaling Technology, #12153), p-Stat3 (Cell Signaling Technology, #9145), E-cadherin (Cell Signaling Technology, #3195), Vimentin (Cell Signaling Technology, #5741), Slug (Cell Signaling Technology, #9585), Zeb1 (Sigma, SAB2102759), Smad3 (Cell Signaling Technology, #9523), Yap1 (Cell Signaling Technology, #14074), CD44 (Abcam, ab51037) and GAPDH (Proteintech, HRP-60004). Membranes were incubated with primary antibody overnight, and with horseradish peroxidase–conjugated secondary antibody for 2 h. SuperSignal Chemiluminescent Substrates (Thermo Fisher Scientific) and imaging systems were used to analysis the results.

### Real-time PCR

RNAiso Plus (Takara) was used to extract total RNAs, which were then reversely transcribed into cDNA using PrimeScript™ RT reagent Kit with gDNA Eraser (TaKaRa) according to the instructions. Real-time PCR was performed using TB Green™ *Premix Ex Taq*™ II (TaKaRa). Primers for VIM (Vimentin) were, forward: 5′-GGTGGACCAGCTAACCAACG-3′ and reverse: 5′-TTGCAGGGTGTTTTCGGCTT-3′; for CDH1 (E-cadherin) were, forward: 5′-GCCATCGCTTACACCATCCTCAG-3′ and reverse: 5′-CTCTCTCGGTCCAGCCCAGTG-3′; for ZEB1 were, forward: 5′-CAGGCAAAGTAAATATCCCTGC-3′ and reverse: 5′-GGTAAAACTGGGGAGTTAGTCA-3′; for SNAI2 (Slug) were, forward: 5′-CTGTGACAAGGAATATGTGAGC-3′ and reverse: 5′-CTAATGTGTCCTTGAAGCAACC-3′. GAPDH was used as endogenous control. Expression difference was analyzed using 2^-ΔΔCT^ method.

### Cell transfection

BC cells were transfected with shRNA lentiviruses, and selected with 2 μg/ml puromycin (Invitrogen) 48 h post-transfection. Cells with gene stable knockdown were maintained in DMEM supplemented with 10% FBS and 2 μg/ml puromycin. MCF7 cells were transfected with FZD7 overexpression lentiviruses and selected with 1 μg/ml puromycin. Cells with FZD7 stable overexpression were cultured in DMEM contained with 10% FBS and 1 μg/ml puromycin.

### Immunofluorescence assay

Cells were grown on cover slips for 24 h, then washed twice with PBS and fixed by 4% paraformaldehyde solution. Cytomembranes were penetrated with 1% Triton-100 solution. Primary antibodies (Cell Signaling Technology, E-cadherin, #3195; Vimentin, #5741) were added at 4 °C overnight. Cells were then incubated with Alexa fluor 488donkey anti-rabbit IgG(H + L) (Invitrogen, A21206) for 2 h, and with DAPI staining solution (Beyotime) for 5 min at room temperature in the dark. The fluorescence was visualized by a confocal microscope.

### Wound healing assay

Cells were cultured in six-well plate. A pipette tip was used to scratch confluent monolayers. Cell debris was washed away with warm PBS. Then cells were cultured for 24 h in serum-free medium. An inverted microscope was used to capture the images.

### Cell invasion assay

Cells were resuspended with serum-free medium and made into 1 × 10^5^/ml. 0.2 ml of the cell suspensions (2 × 10^4^ cells) were added to the upper chamber of transwell plates (Corning Costar). In the lower chamber, 0.5 ml medium with 10% FBS was added to promote cell movement through the pores of the membrane. After 24 h, the cotton swab was used to clean the inside of the chamber. Migrated cells were fixed with a paraformaldehyde solution for 15 min and stained with the Crystal Violet. Images were captured using an inverted microscope.

### Mammosphere assay

1 × 10^4^ cells were seeded in 6-well Ultra-Low Attachment Surface Polystyrene culture plates (Corning Costar) added with complete MammoCult™ Human Medium (STEMCELL Technologies). Cells were cultured at 37 °C and 5% CO_2_ for 8 days. Analysis was performed by counting the number of mammospheres in 5 randomly selected fields under an inverted microscope.

### Flow cytometry

The proportion of Lgr5^+^ stem cells was determined by flowcytometry. Pre-cooled stain buffer (Invitrogen) was used to wash and resuspend cells. 1 × 10^6^ cells were stained with 5 μl PE-conjugated anti-hLgr5 (Invitrogen) antibody on ice for 30 min. Stained populations were sorted and analyzed on BD Accuri C6 Plus (BD Biosciences).

### In vivo study

All animal experiments were performed using 8-week-old NOD SCID mice. Mice were maintained in laminar flow rooms with constant temperature and humidity. 5 × 10^5^ MDA-MB-231 cells transfected with FZD7 lentiviruses or control lentiviruses were injected into the fat pad of mice. Tumor growth was followed every 7 days by tumor diameter measurements using vernier calipers. Tumor volumes (V) were calculated using 1/2 × length×width^2^. Ten weeks after inoculation, tumors and lungs were obtained, and fixed in 4% paraformaldehyde solution. Lung samples were stained with HE and images were captured by using a positive microscope. All animal experiments were approved by the ethics committee of the China Medical University.

### Immunohistochemistry

Immunohistochemistry (IHC) analysis was performed on 4 μm paraffin sections of human BC tissues and mouse xenograft tumors. Xylene and gradient alcohols were performed to deparaffinize and hydrate, respectively. 3% H_2_O_2_ was added to the sections to remove endogenous peroxidase. Sections were incubated with citrate buffer to repair antigen, and blocked by BSA. Primary antibodies were used as follows: Fzd7 (Santa Cruz Biotechnology, sc-293,261), Col6a1 (Santa Cruz Biotechnology, sc-377,143), Ki67 (Invitrogen, 14,569,982), Cleaved caspase-3 (Cell Signaling Technology, #9661). After incubated with primary antibodies overnight at 4 °C in a wet box, biotinylated secondary antibodies were added. Diaminobenzidine (BOSTER) was used to stain the sections dissolved in Tris-HCl and H_2_O_2_. Then sections were re-stained in hematoxylin, dehydrated with gradient alcohol and xylene and sealed with cover slides.

### Human specimens

Paraffin-embedded BC tissues including 10 invasive ductal BC (IDC), 5 invasive lobular BC (ILC) and 3 ductal BC in situ (DCIS) were obtained from Liaoning Province Tumor Hospital with the informed consent of the patients. The use of these specimens for research purposes was approved by Institutional Research Ethics Committee of China Medical University.

### Co-immunoprecipitation

Cell lysates of MDA-MB-231 and Hs578T were centrifuged at 12000 rpm at 4 °C. Fifty microliter supernatant was taken as normal sample and 20 μl protein A/G agarose beads (Santa Cruz Biotechnology, sc-2003) were added to the remaining supernatant and incubated for 30 min. After incubating and centrifuging, samples were divided into two tubes. Two microgram Fzd7 (R&D systems) and 2 μg mouse IgG (Santa Cruz Biotechnology) were added, respectively. Each tube sample was combined with 20 μl protein A/G agarose beads overnight. The non-specifically bound proteins were removed by washing the agarose beads. Following procedures were same as Western blot.

### Statistical analysis

GraphPad Prism 7.0 was used to analyze the data, and all data were presented as the mean ± s.e.m. Comparison of means within two groups was analyzed using two-tailed unpaired Student’s *t* test. *P* < 0.05 was considered as significant.

## Results

### Fzd7 is associated with mesenchymal phenotype

**Fig. 1:**
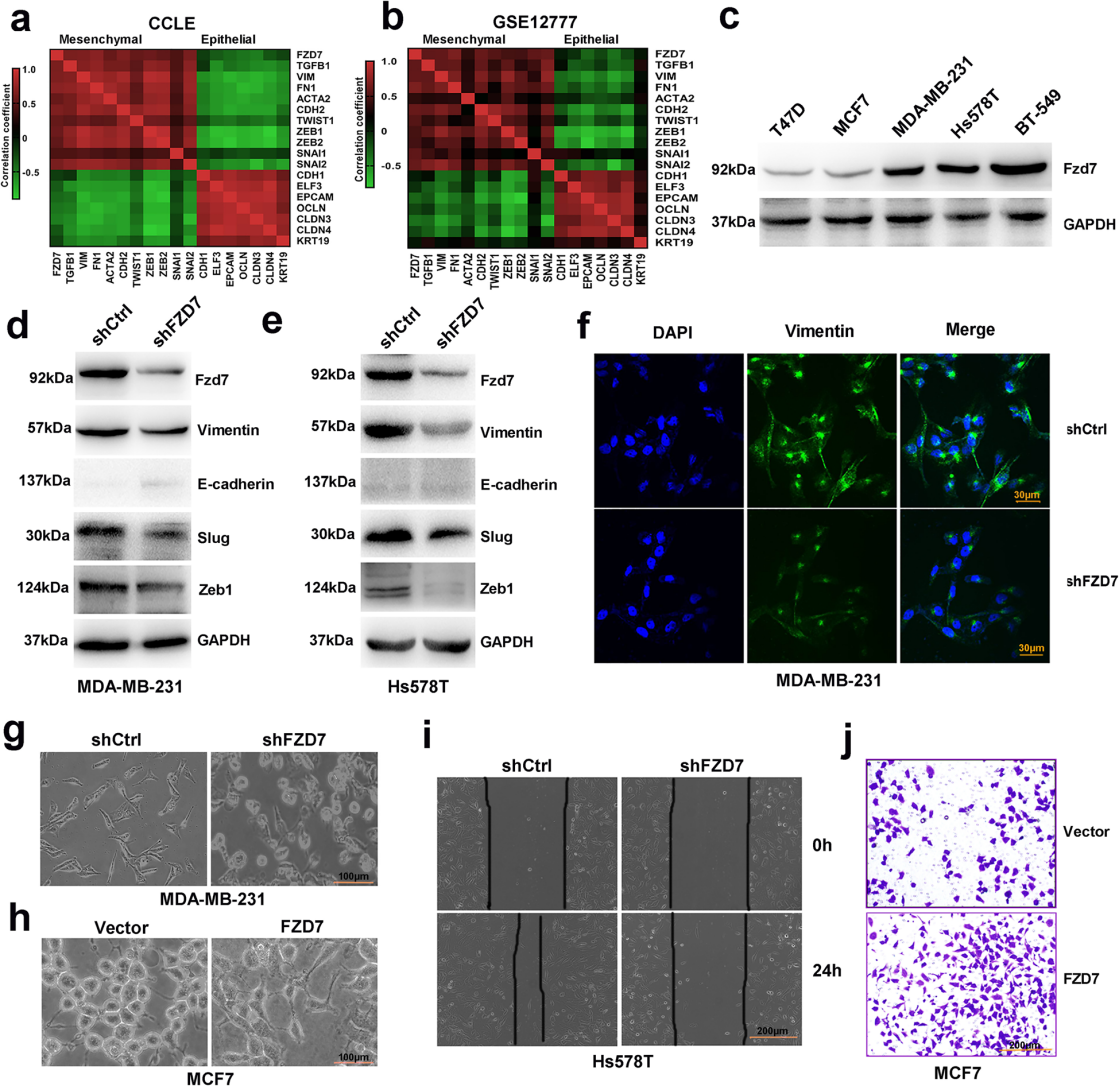
Fzd7 is associated with mesenchymal phenotype. **a** and **b** Heat maps generated from CCLE database (**a**) and GSE12777 database (**b**) demonstrated the correlation of *FZD7* with mesenchymal-related genes and epithelial-related genes in human BC cell lines. **c** Expression of Fzd7 was detected in some representative cell lines by Western blot. **d** and **e** Expression of Fzd7, Vimentin, E-cadherin, Slug and Zeb1 was detected in MDA-MB-231 cells (**d**) and Hs578T cells (**e**) transfected with shCtrl or shFZD7 by Western blot. **f** Expression of Vimentin was detected in MDA-MB-231 cells transfected with shCtrl or shFZD7 by Immunofluorescence staining. **g** Morphology of MDA-MB-231 cells transfected with shCtrl or shFZD7 was shown. **h** Morphology of MCF7 cells transfected with control vector or FZD7 overexpression vector was shown. **i** Migration of Hs578T cells transfected with shCtrl or shFZD7 was analyzed by Wound healing. **j** Invasion of MCF7 cells transfected with control vector or FZD7 overexpression vector was analyzed by Transwell. All experiments were carried out three times

To investigate the association of Fzd7 with BC cell mesenchymal phenotype, we first interrogated Cancer Cell Line Encyclopedia (CCLE) database and GSE12777 database. It was shown that expression of FZD7 was positively correlated with that of mesenchymal-related genes, whereas negatively correlated with that of epithelial-related genes, in human BC cell lines (Fig. 1a-b, Supplementary file 1: Fig. S1A-B). Expression of Fzd7 in some representative cell lines was subsequently determined. As shown by Western blot detection, Fzd7 was highly expressed in several mesenchymal-like cell lines such as MDA-MB-231, Hs578T and BT-549 (Fig. 1c).

To further explore the role of Fzd7 in BC cell mesenchymal phenotype, MDA-MB-231 cells and Hs578T cells were transfected with FZD7 shRNA lentiviruses. Fzd7 knockdown significantly reduced expression of Vimentin, N-cadherin and EMT transcription factor (EMT-TF) Slug and Zeb1 (Fig. 1d-e, Supplementary file 1: Fig. S1C-F). The inhibitory effect of Fzd7 knockdown on expression of Vimentin was also confirmed by Immunofluorescent staining (Fig. 1f). Consistently, Fzd7 overexpression in MCF7 cells upregulated expression of Vimentin, Slug and Zeb1 (Supplementary file 1: Fig. S2A-C). Fzd7 knockdown induced an epithelial-like morphology in MDA-MB-231 cells, while Fzd7 overexpression induced a mesenchymal-like morphology in MCF7 cells (Fig. 1g-h). Moreover, Fzd7 knockdown suppressed BC cell migration and invasion, whereas Fzd7 overexpression promoted BC cell motility (Fig. 1i-j, Supplementary file 1: Fig. 3a-d).

### Fzd7 contributes to BC cell stemness

**Fig. 2:**
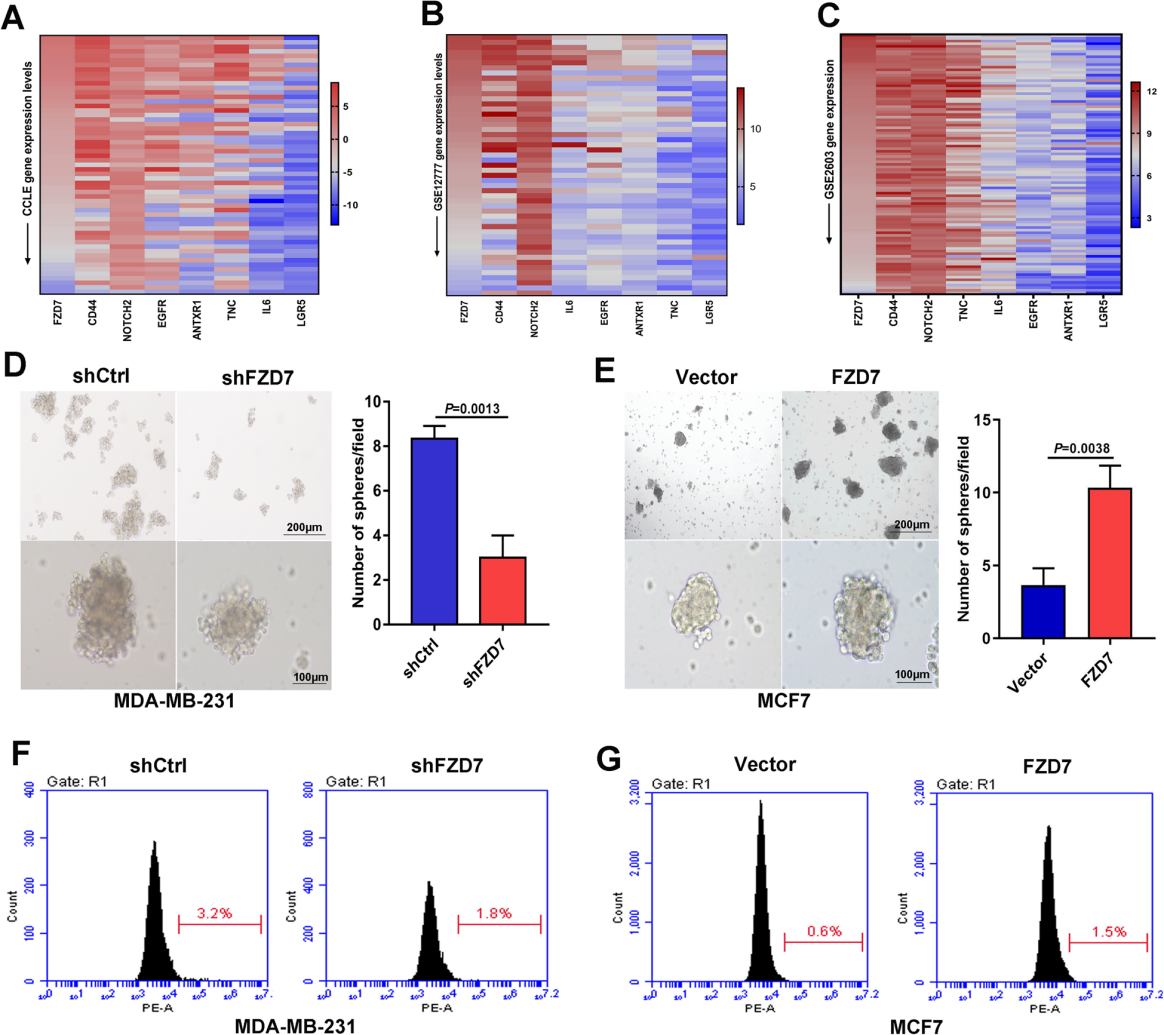
Fzd7 contributes to BC cell stemness. **a** and **b** Heat maps generated from CCLE database (**a**) and GSE12777 database (**b**) demonstrated the correlation of *FZD7* with stemness-related genes in human BC cell lines. **c** Heat map generated from GSE2603 database demonstrated the correlation of *FZD7* with stemness-related genes in human BC tissues. **d** Mammosphere formation in MDA-MB-231 cells transfected with shCtrl or shFZD7 was shown. **e** Mammosphere formation in MCF7 cells transfected with control vector or FZD7 overexpression vector was shown. **f** The fraction of Lgr5^+^ subpopulation in MDA-MB-231 cells transfected with shCtrl or shFZD7 was determined by flowcytometry. **g** The fraction of Lgr5^+^ subpopulation in MCF7 cells transfected with control vector or FZD7 overexpression vector was determined by flowcytometry. All experiments were carried out three times. Data are expressed as Mean ± s.e.m

To explore whether Fzd7 was related to BC cell stemness, we interrogated CCLE database, and found a correlation of FZD7 with a panel of stemness-related genes including CD44, LGR5, NOTCH2, EGFR, IL6 as well as TNC [18] and ANTXR1 [19] (Fig. 2a, Supplementary file 1: Fig. S4A). Interrogation of GSE12777 revealed that FZD7 was significantly correlated with CD44, NOTCH2, EGFR, IL6 and ANTXR1 (Fig. 2b, Supplementary file 1: Fig. S4B). Moreover, interrogation of GSE2603 indicated that FZD7 was correlated with LGR5, NOTCH2, EGFR and *TNC* in a panel of human BC tissues (Fig. 2c, Supplementary file 1: Fig. S4C).

Mammosphere formation test was then used to evaluate the role of Fzd7 in BC cell stemness. Fzd7 knockdown impaired mammosphere formation capacity of MDA-MB-231 cells and Hs578T cells, while Fzd7 overexpression promoted mammosphere formation in MCF7 cells (Fig. 2d-e, Supplementary file 1: Fig. S5A). Furthermore, Fzd7 knockdown reduced the fraction of Lgr5^+^ subpopulation in MDA-MB-231 cells and Hs578T cells, whereas Fzd7 overexpression increased the fraction of Lgr5^+^ subpopulation in MCF7 cells (Fig. 2f-g, Supplementary file 1: Fig. S5B). Loss and gain of Fzd7 also affected CD44 expression in BC cells (Supplementary file 1: Fig. S5C-D). Moreover, Fzd7 knockdown had no effect on the subpopulation of aldehyde dehydrogenase 1 (ALDH1) which was recognized as a marker of epithelial-like stemness [11] (data not shown).

### Fzd7 knockdown suppresses BC tumorigenesis and metastasis

**Fig. 3:**
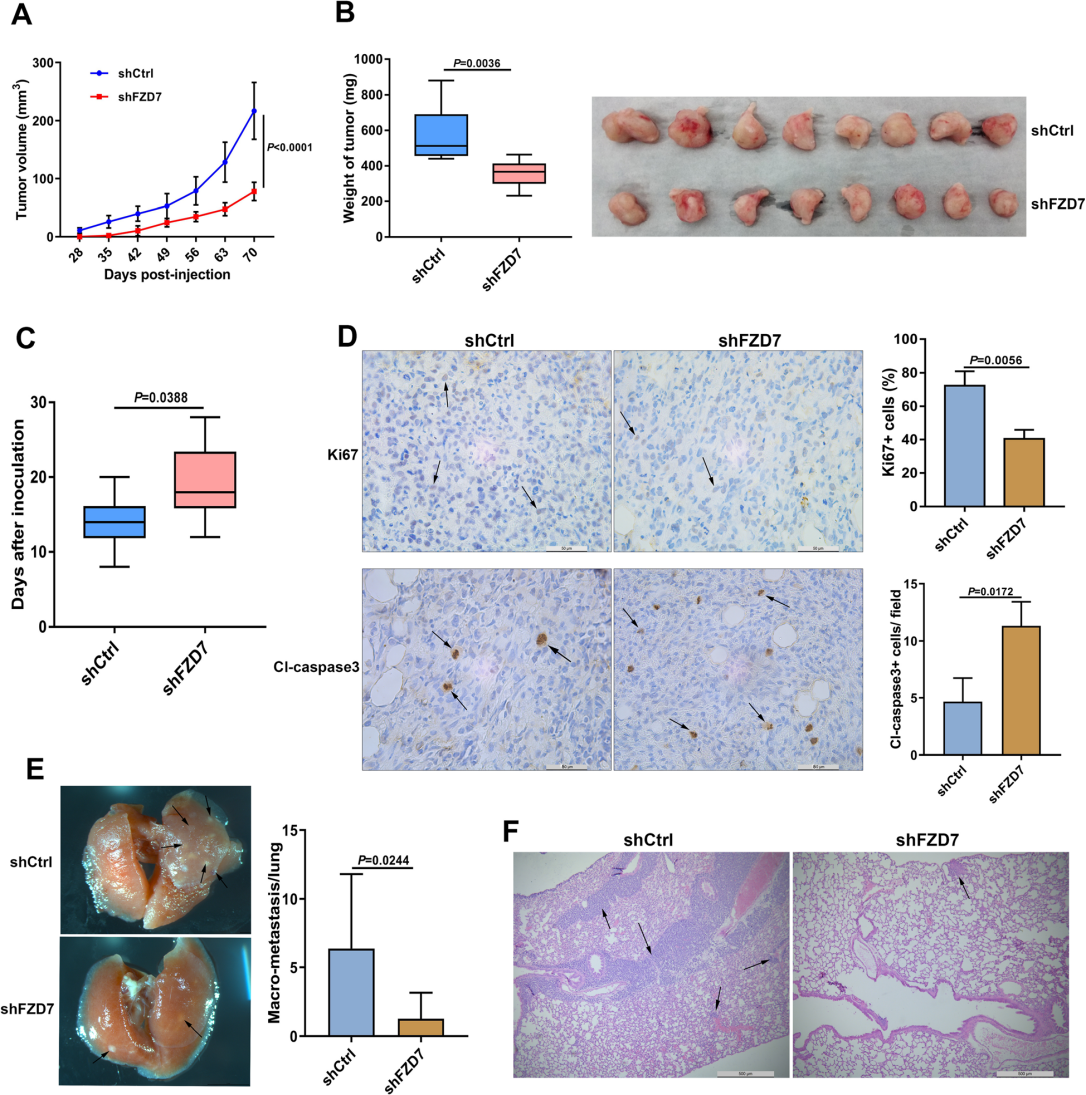
Fzd7 knockdown suppresses BC tumorigenesis and metastasis. **a** Growth curves of xenograft tumors derived from MDA-MB-231 cells transfected with shCtrl or shFZD7 were shown (8 mice in each group). **b** Tumors were harvested and weighed 10 weeks post-inoculation. **c** Latent periods of xenograft tumors were compared between two groups. **d** Expression of Ki67 and cleaved caspase-3 (Cl-caspase3) in xenograft tumors was determined by Immunohistochemistry. Ki67-positive cells and cleaved caspase-3 -positive cells were quantified. **e** Macro-metastasis on mouse lung surface was shown. The numbers of lung macro-metastasis were counted. **f** Lung metastasis was shown by HE staining. Data are expressed as Mean ± s.e.m

To evaluate the effect of Fzd7 knockdown on BC tumorigenesis and metastasis in vivo, NOD SCID mice were inoculated with MDA-MB-231 cells transfected with FZD7 shRNA lentiviruses or control shRNA lentiviruses. Xenograft tumors were harvested and lung metastasis was analyzed 10 weeks after inoculation. Tumors derived from FZD7 shRNA-transfected cells grew much slower than those from control shRNA-transfected cells, indicating that Fzd7 knockdown inhibited tumor growth (Fig. 3a-b). Moreover, Fzd7 knockdown delayed the appearance of xenograft tumors (Fig. 3c). Tumors derived from FZD7 shRNA-transfected cells exhibited less Ki67-positive cells but more Cleaved caspase-3-positive cells (Fig. 3d). Macro-metastasis on lung surface was found in 6 of 8 control mice, and in 3 of 8 mice inoculated with FZD7 shRNA-transfected cells (Fig. 3e). In mice without macro-metastasis, micro-metastasis existed in 2 control mice and 1 mouse inoculated with FZD7 shRNA-transfected cells (Fig. 3f).

### Fzd7 modulates non-canonical Wnt pathway

**Fig. 4:**
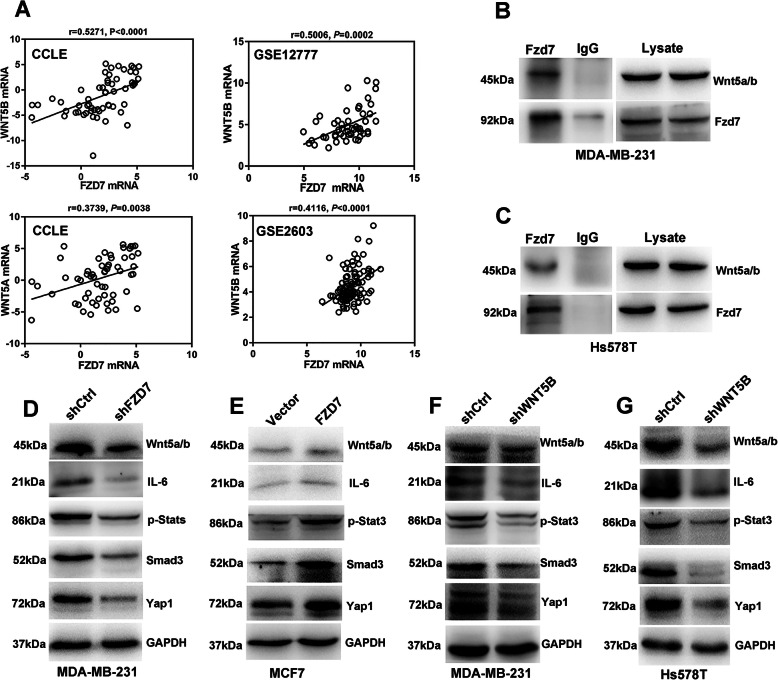
Fzd7 modulates non-canonical Wnt pathways. **a** Interrogation of databases showed the correlation of *FZD7* with *WNT5* in BC cell lines (CCLE and GSE12777) and tissues (GSE2603). **b** and **c** Co-IP test was performed to verify the binding of Wnt5a/b to Fzd7 in MDA-MB-231 cells (**b**) and Hs578T cells (**c**). **d** Expression of Wnt5a/b, IL-6, p-Stat3, Smad3 and Yap1 was detected in MDA-MB-231 cells transfected with shCtrl or shFZD7 by Western blot. **e** Expression of Wnt5a/b, IL-6, p-Stat3, Smad3 and Yap1 was detected in MCF7 cells transfected with control vector or FZD7 overexpression vector by Western blot. **f** and **g** Expression of Wnt5a/b, IL-6, p-Stat3, Smad3 and Yap1 was detected in MDA-MB-231 cells (**f**) and Hs578T cells (**g**) transfected with shCtrl or shWNT5B by Western blot. All experiments were carried out three times. Data are expressed as Mean ± s.e.m

We next inquired the potential Wnt ligand binding to Fzd7. Intriguingly, a positive correlation of FZD7 with WNT5, especially WNT5B, was identified in both BC cell lines and BC tissues (Fig. 4a), suggesting Fzd7 might initiate non-canonical Wnt pathways. Subsequent Co-IP test confirmed the binding of Wnt5a/b to Fzd7 in both MDA-MB-231 cells and Hs578T cells (Fig. 4b-c). Furthermore, Fzd7 knockdown reduced expression of Wnt5a/b in MDA-MB-231 cells (Fig. 4d). In contrast, Fzd7 upregulation induced expression of Wnt5a/b in MCF7 cells (Fig. 4e). Loss and gain of Fzd7 affected expression of several intracellular oncogenic molecules including phosphorylated Stat3 (p-Stat3), Smad3 and Yes-associated protein 1 (Yap1) (Fig. 4d-e). Similar to Fzd7 knockdown, Wnt5b knockdown downregulated these molecules in MDA-MB-231 cells and Hs578T cells (Fig. 4f-g).

### Wnt5b is involved in BC cell mesenchymal phenotype and stemness

**Fig. 5:**
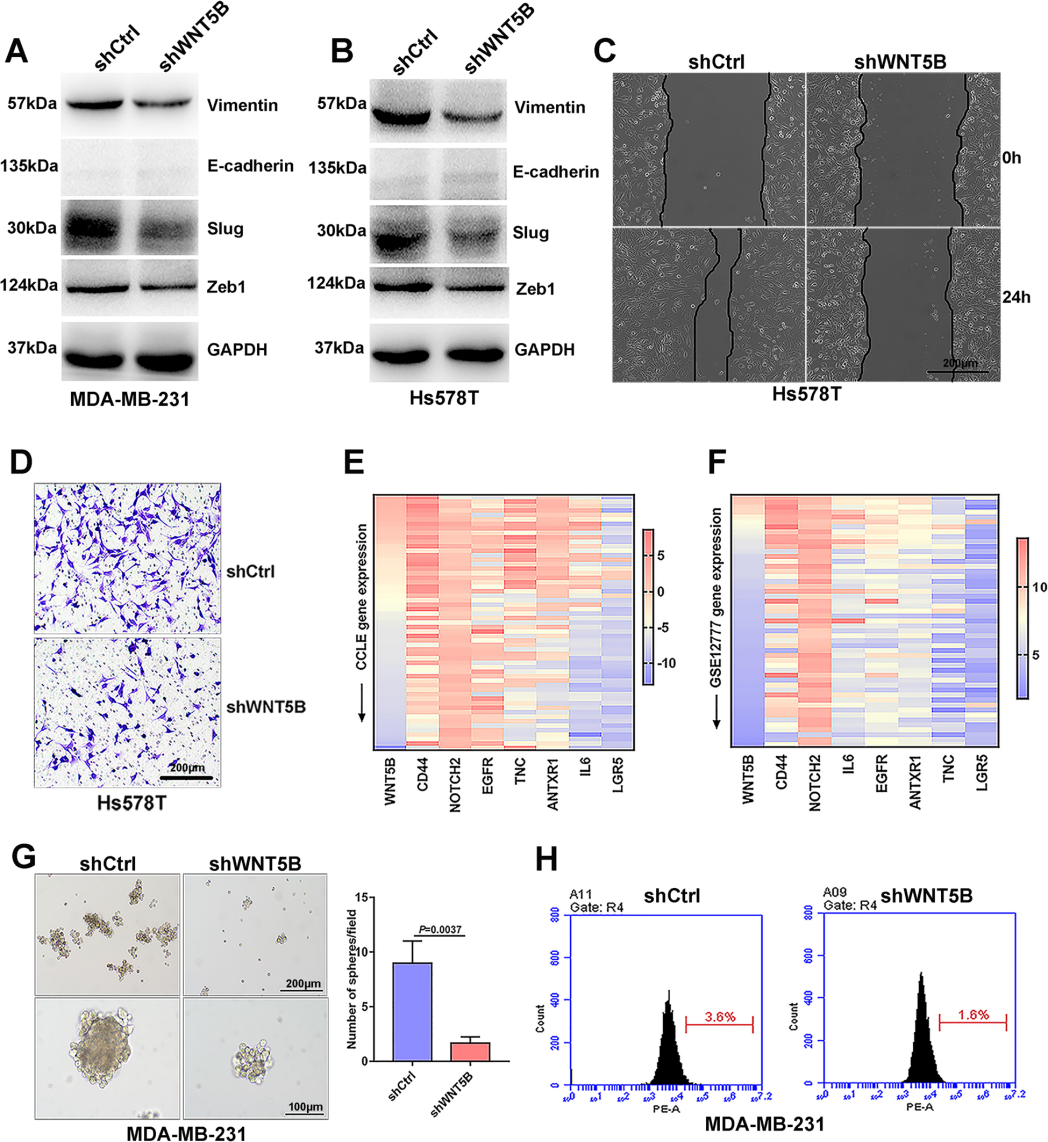
Wnt5b is involved in BC cell mesenchymal phenotype and stemness. **a** and **b** Expression of Vimentin, E-cadherin, Slug and Zeb1 was detected in MDA-MB-231 cells (**a**) and Hs578T cells (**b**) transfected with shCtrl or shWNT5B by Western blot. **c** and **d** Migration (**c**) and invasion (**d**) of Hs578T cells transfected with shCtrl or shWNT5B were analyzed by Wound healing or Transwell. **e** and **f** Heat map generated from CCLE database (**e**) and GSE12777 database (**f**) demonstrated the correlation of *WNT5B* with stemness-related genes in human BC cell lines. **g** Mammosphere formation in MDA-MB-231 cells transfected with shCtrl or shWNT5B was shown. **h** The fraction of Lgr5^+^ subpopulation in MDA-MB-231 cells transfected with shCtrl or shWNT5B was determined by flowcytometry. All experiments were carried out three times. Data are expressed as Mean ± s.e.m

Since Wnt5b was a potential ligand for Fzd7, we subsequently determined the role of Wnt5b in BC cell mesenchymal phenotype and stemness. Interrogation of databases showed that WNT5B, similar to FZD7, was positively associated with mesenchymal-related genes, whereas negatively associated with epithelial-related genes (Supplementary file 1: Fig. S6A-B). Wnt5b knockdown reduced expression of Vimentin, Slug and Zeb1 in BC cells (Fig. 5a-b, Supplementary file 1: Fig. S6C-D). Moreover, Wnt5b knockdown suppressed BC cell migration and invasion (Fig. 5c-d). Interrogation of databases also showed that WNT5B was associated with the panel of stemness-related genes (Fig. 5e-f). Consistently, Wnt5b knockdown impaired mammosphere formation capacity, reduced the fraction of Lgr5^+^ subpopulation and suppressed expression of CD44 in BC cells (Fig. 5g-h, Supplementary file 1: Fig. S7A-C).

### Col6a1 is implicated in Fzd7 signaling

**Fig. 6:**
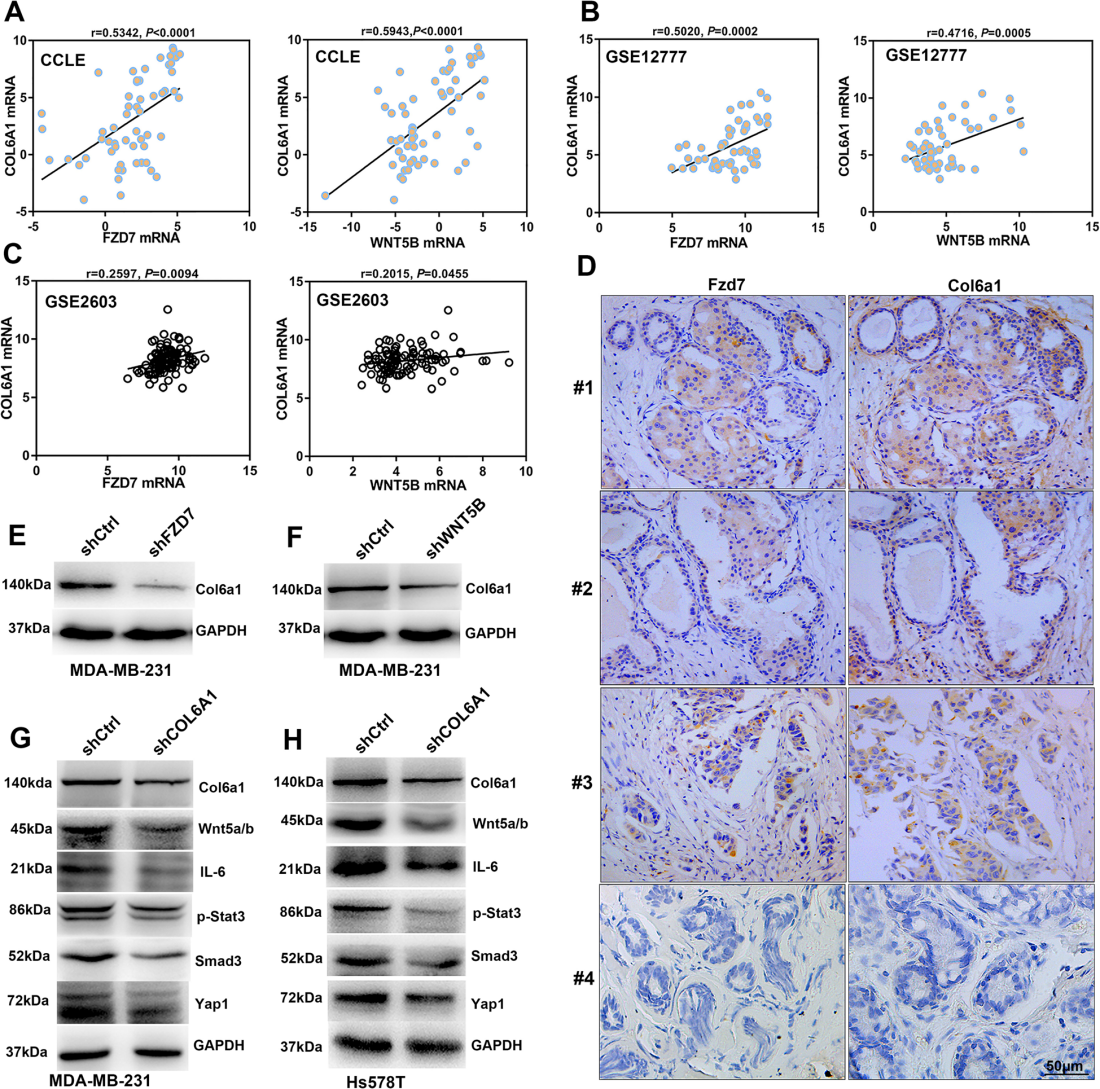
Col6a1 is implicated in Fzd7 signaling. **a** and **b** Interrogation of CCLE database (**a**) and GSE12777 database (**b**) showed the correlation of *FZD7* and *WNT5B* with *COL6A1* in BC cell lines. **c** Interrogation of GSE2603 database showed the correlation of *FZD7* and *WNT5B* with *COL6A1* in BC tissues. **d** Expression of Fzd7 and Col6a1 in BC tissues was detected by Immunohistochemistry. #1, #2, #3 and #4 are representative specimens. **e** Expression of Col6a1 was detected in MDA-MB-231 cells transfected with shCtrl or shFZD7 by Western blot. **f** Expression of Col6a1 was detected in MDA-MB-231 cells transfected with shCtrl or shWNT5B by Western blot. **g** and **h** Expression of Col6a1, Wnt5a/b, IL-6, p-Stat3, Smad3 and Yap1 was detected in MDA-MB-231 cells (**g**) and Hs578T cells (**h**) transfected with shCtrl or shCOL6A1 by Western blot. All experiments were carried out three times

It was demonstrated that collagen signaling contributed to BC stemness and metastasis [19–21]. By interrogating databases, we identified the implication of Col6a1 in Fzd7 signaling. It was revealed that COL6A1 was associated with FZD7 and WNT5B in both BC cell lines and BC tissues (Fig. 6a-c). Immunohistochemistry detection revealed that Fzd7 and Col6a1 were expressed in a similar pattern in BC tissues (Fig. 6d). Expression of Col6a1 was downregulated in MDA-MB-231 cells with either Fzd7 knockdown or Wnt5b knockdown (Fig. 6e-f). Intriguingly, Col6a1 knockdown inhibited expression of Wnt5a/b in BC cells (Fig. 6g-h). Col6a1 knockdown also reduced expression of p-Stat3, Smad3 and Yap1 (Fig. 6g-h).

### Col6a1 maintains BC cell mesenchymal phenotype and stemness

**Fig. 7:**
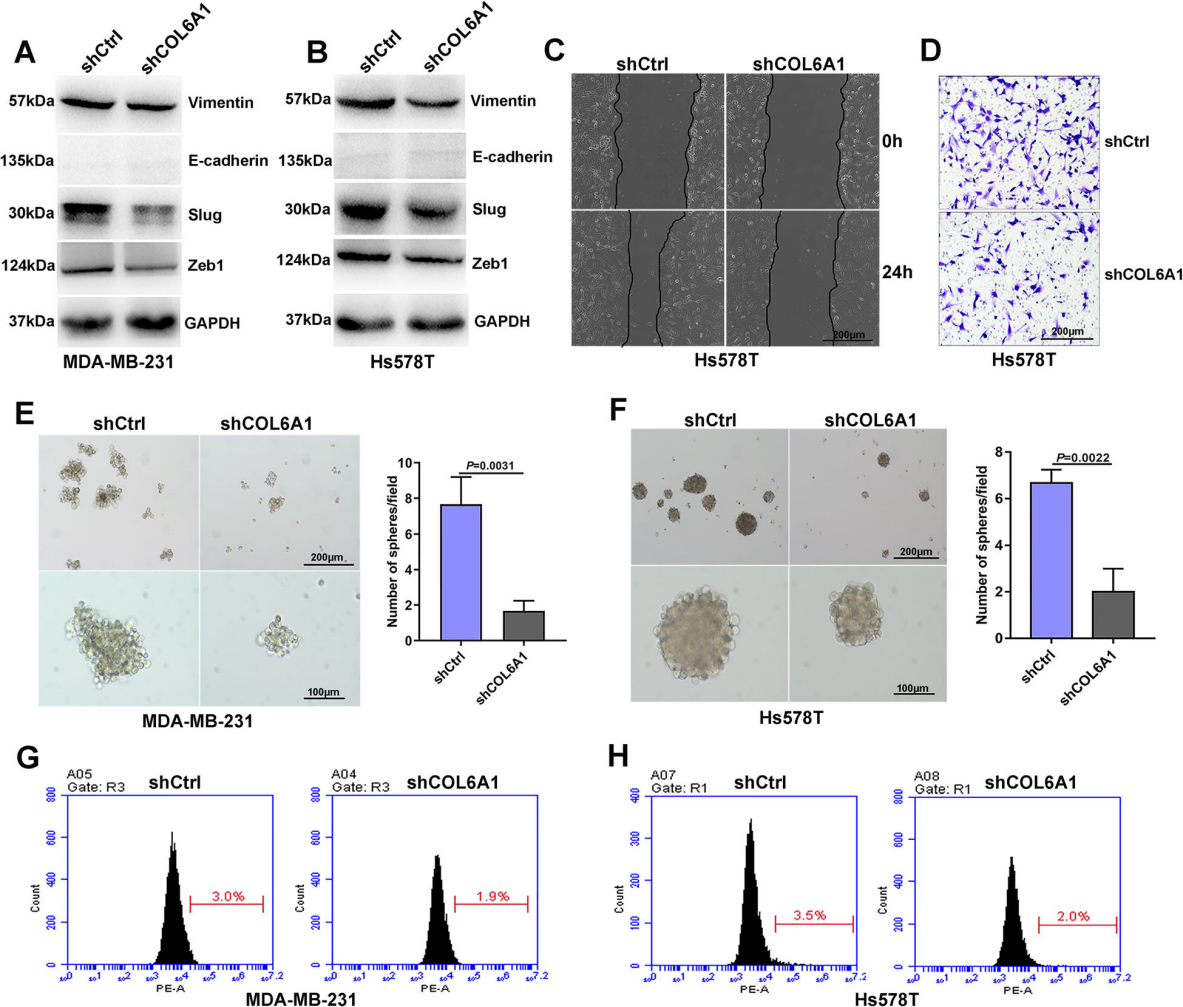
Col6a1 maintains BC cell mesenchymal phenotype and stemness. **a** and **b** Expression of Vimentin, E-cadherin, Slug and Zeb1 was detected in MDA-MB-231 cells (**a**) and Hs578T cells (**b**) transfected with shCtrl or shCOL6A1 by Western blot. **c** and **d** Migration (**c**) and invasion (**d**) of Hs578T cells transfected with shCtrl or shCOL6A1 were analyzed by Wound healing or Transwell. **e** and **f** Mammosphere formation in MDA-MB-231 cells (**e**) and Hs578T cells (**f**) transfected with shCtrl or shCOL6A1 was shown. **g** and **h** The fraction of Lgr5^+^ subpopulation in MDA-MB-231 cells (**g**) and Hs578T cells (**h**) transfected with shCtrl or shCOL6A1 was determined by flowcytometry. All experiments were carried out three times. Data are expressed as Mean ± s.e.m

We finally assessed the role of Col6a1 in BC cell mesenchymal phenotype and stemness. Interrogation of databases showed that COL6A1 was positively associated with mesenchymal-related genes, whereas negatively associated with epithelial-related genes (Supplementary file 1: Fig. S8A-B). Col6a1 knockdown reduced expression of Vimentin, Slug and Zeb1 in BC cells (Fig. 7a-b, Supplementary file 1: Fig. S8C-D). Col6a1 knockdown inhibited BC cell migration and invasion (Fig. 7c-d). Interrogation of databases further showed that COL6A1 was also associated with the panel of stemness-related genes (Supplementary file 1: Fig. S8E-F). As expected, Col6a1 knockdown impaired mammosphere formation capacity of BC cells (Fig. 7e-f). Moreover, Col6a1 knockdown reduced the fraction of Lgr5^+^ subpopulation (Fig. 7g-h).

## Discussion

MMTV-*Wnt1*, MMTV-*Wnt10b* and MMTV-*βcatΔN* transgenic mice have clearly demonstrated that canonical Wnt/β-catenin pathway has the capacity to induce BC development [22]. In human BC, Wnt10b, but not Wnt1, as well as Fzd7 and Wnt co-receptor LDL receptor-related proteins 6 (LRP6) was overexpressed in TNBC, with activation of β-catenin pathway [7, 23–25]. However, our study revealed that Fzd7-mediated non-canonical Wnt pathway contributed to BC EMT and stemness. Intriguingly, both Wnt5a/b and Fzd7 were overexpressed in MMTV-*Wnt1* tumors, suggesting that non-canonical Fzd7 pathway was involved in Wnt1-induced BC [26]. In Her2^+^ metastasis-initiating cells, both canonical and non-canonical Wnt ligands were responsible for EMT-like program and early dissemination [27]. Therefore, we conclude that Fzd7 may mediate both β-catenin-dependent and β-catenin-independent pathways in human BC.

Interrogation of databases indicated that Fzd7-Wnt5b pathway cross-talked with oncogenic pathways such as IL-6, EGFR, NOTCH and TGF-β1. We further revealed that Fzd7-Wnt5b signaling modulated several key intracellular molecules involved in these pathways including p-Stat3, Smad3 and Yap1. These findings uncovered the molecular mechanism underlying Fzd7-induced EMT and stemness. Studies have shown that IL-6, EGFR, NOTCH and TGF-β1 form an oncogenic signal network in cancer initiation and progression [15, 17, 28–30]. Our study added non-canonical Fzd7 and Wnt5b into this network. Notably, Fzd7 signaling modulated Wnt5a/b expression, forming an autocrine positive feedback. Stat3, Smad3 and Yap1 might be responsible for this induction [31–33].

Lgr5 is both a marker of adult stem cells and a modulator of their activity [34]. Lgr5^+^ mammary epithelial cells were shown sufficient and necessary for postnatal mammary organogenesis [35]. Single-cell analysis demonstrated that early metastatic BC cells highly expressed EMT and stemness -associated genes including *LGR5* [36]. BC cell-derived Tenascin C (TNC) promoted Lgr5 expression, stemness and pulmonary metastasis [18]. Interrogation of CCLE and GSE2603 showed that *FZD7* was associated with *TNC* and *LGR5* in both BC cell lines and BC tissues. Furthermore, our study indicated that Fzd7 expression modulated Lgr5^+^ subpopulation. In consistent with our finding, Fzd7 was revealed enriched in Lgr5^+^ intestinal stem cells. Deletion or blocking of Fzd7 reduced the number of stem cells or impaired the function of stem cells [37, 38]. Moreover, Wnt5a was shown to stimulate Lgr5 expression in osteoblasts [39].

Increasing evidence has indicated that collagen signaling promotes BC stemness and metastasis. Discoidin Domain Receptor 1 (DDR1), when exposed to its ligand Collagen I, induced BC cell stemness and lung metastasis in a Stat3-dependent manner [21]. Interrogation of databases showed that *FZD7* was closely correlated with *COL1A1* (data not shown). Activation of ANTXR1 by its ligand C5a, a fragment of Col6a3, also increased BC cell stemness and lung metastasis [19]. Interrogation of databases showed that *FZD7* was associated with *ANTXR1*. These observations, in combine with our finding that Fzd7-Wnt5b modulated expression of Col6a1, indicate that non-canonical Fzd7 signaling cross-talked with Collagen signaling.

## Conclusions

Our study identified that Fzd7 contributed to BC cell EMT and stemness, inducing tumorigenesis and metastasis, via a non-canonical Wnt5b pathway. We also discovered that Col6a1 was implicated in Fzd7-Wnt5b signaling, and mediated Fzd7-Wnt5b -induced mesenchymal-like stemness. These findings linked non-canonical Wnt pathway to Collagen signaling in BC progression. Mechanistically, we proposed an oncogenic network consisting of non-canonical Wnt, Collagen, IL-6/Stat3, TGF-β1/Smad3, EGFR and NOTCH2.

## Supplementary information

**Additional file 1: Supplementary Figure S1.** Interrogation of CCLE database (A) and GSE12777 database (B) showed the correlation of FZD7 with TGFB1, VIM, SNAI2 (Slug) and ZEB1 in BC cell lines. Expression of VIM, CDH1, SNAI2 and ZEB1 was detected in MDA-MB-231 (C) and Hs578T (D) cells transfected with shCtrl or shFZD7 by real-time PCR. Expression of N-cadherin was detected in MDA-MB-231 (E) and Hs578T (F) cells transfected with shCtrl or shFZD7 by Western blot. All experiments were carried out three times. Data are expressed as Mean ± s.e.m. **Supplementary Figure S2.** (A) Expression of Fzd7, Vimentin, E-cadherin, Slug and Zeb1 was detected in MCF7 cells transfected with control vector or FZD7 overexpression vector by Western blot. Expression of Vimentin (B) and E-cadherin (C) was detected in MCF7 cells transfected with control vector or FZD7 overexpression vector by Immunofluorescence staining. All experiments were carried out three times. **Supplementary Figure S3.** Migration (A) and Invasion (B) of MDA-MB-231 cells transfected with shCtrl or shFZD7 was analyzed by Wound healing and Transwell, respectively. (C) Invasion of Hs578T cells transfected with shCtrl or shFZD7 was analyzed by Transwell. (D) Migration of MCF7 cells transfected with control vector or FZD7 overexpression vector was analyzed by Wound healing. All experiments were carried out three times. **Supplementary Figure S4.** (A) Interrogation of CCLE database showed the correlation of FZD7 with CD44, LGR5, EGFR and NOTCH2 in BC cell lines. (B) Interrogation of GSE12777 database showed the correlation of FZD7 with CD44, EGFR and NOTCH2 in BC cell lines. (C) Interrogation of GSE2603 database showed the correlation of FZD7 with LGR5, EGFR and NOTCH2 in BC tissues. **Supplementary Figure S5.** (A) Mammosphere formation in Hs578T cells transfected with shCtrl or shFZD7 was shown. (B) The fraction of Lgr5^+^ subpopulation in Hs578T cells transfected with shCtrl or shFZD7 was determined by flowcytometry. (C) Expression of CD44 was detected in MDA-MB-231 cells transfected with shCtrl or shFZD7 by Western blot. (D) Expression of CD44 was detected in MCF7 cells transfected with control vector or FZD7 overexpression vector by Western blot. All experiments were carried out three times. Data are expressed as Mean ± s.e.m. **Supplementary Figure S6.** Heat maps generated from CCLE database (A) and GSE12777 database (B) demonstrated the correlation of WNT5B with mesenchymal-related genes and epithelial-related genes in human BC cell lines. Expression of VIM, CDH1, SNAI2 and ZEB1 was detected in MDA-MB-231 (C) and Hs578T (D) cells transfected with shCtrl or shWNT5B by real-time PCR. All experiments were carried out three times. Data are expressed as Mean ± s.e.m. **Supplementary Figure S7.** (A) Mammosphere formation in Hs578T cells transfected with shCtrl or shWNT5B was shown. (B) The fraction of Lgr5^+^ subpopulation in Hs578T cells transfected with shCtrl or shWNT5B was determined by flowcytometry. (C) Expression of CD44 was detected in MDA-MB-231 cells transfected with shCtrl or shWNT5B by Western blot. All experiments were carried out three times. Data are expressed as Mean ± s.e.m. **Supplementary Figure S8.** Heat maps generated from CCLE database (A) and GSE12777 database (B) demonstrated the correlation of COL6A1 with mesenchymal-related genes and epithelial-related genes in human BC cell lines. Expression of VIM, CDH1, SNAI2 and ZEB1 was detected in MDA-MB-231 (C) and Hs578T (D) cells transfected with shCtrl or shCOL6A1 by real-time PCR. Heat maps generated from CCLE database (E) and GSE12777 database (F) demonstrated the correlation of COL6A1 with stemness-related genes in human BC cell lines. All experiments were carried out three times. Data are expressed as Mean ± s.e.m.

## Abbreviations

EMT: Epithelial-mesenchymal transition
BC: Breast cancer
Fzd7: Frizzled 7
p-STAT3: Phosphorylated Stat3
CRD: Cysteine-rich domain
HCC: Hepatocellular carcinoma.
TNBC: Triple-negative breast cancer
Col6a1: Collagen
type VI: Alpha 1
CCLE: Cancer Cell Line Encyclopedia
EMT-TF: EMT transcription factor
ALDH1: Aldehyde dehydrogenase 1
Yap1: Yes-associated protein 1
TNC: Tenascin C
LRP6: LDL receptor-related proteins 6
DDR1: Discoidin Domain Receptor 1
