# Supplementary material for: Non-canonical Fzd7 signaling contributes to breast cancer mesenchymal-like stemness involving Col6a1
Source: Cell Commun Signal. 2020 Sep 7;18:143. doi: 10.1186/s12964-020-00646-2 (PMC7487719; doi:10.1186/s12964-020-00646-2)
Supplement: Supplementary file 2 — Additional file 1: Supplementary Figure S1. Interrogation of CCLE database (A) and GSE12777 database (B) showed the correlation of FZD7 with TGFB1, VIM, SNAI2 (Slug) and ZEB1 in BC cell lines. Expression of VIM, CDH1, SNAI2 and ZEB1 was detected in MDA-MB-231 (C) and Hs578T (D) cells transfected with shCtrl or shFZD7 by real-time PCR. Expression of N-cadherin was detected in MDA-MB-231 (E) and Hs578T (F) cells transfected with shCtrl or shFZD7 by Western blot. All experiments were carried out three times. Data are expressed as Mean ± s.e.m. Supplementary Figure S2. (A) Expression of Fzd7, Vimentin, E-cadherin, Slug and Zeb1 was detected in MCF7 cells transfected with control vector or FZD7 overexpression vector by Western blot. Expression of Vimentin (B) and E-cadherin (C) was detected in MCF7 cells transfected with control vector or FZD7 overexpression vector by Immunofluorescence staining. All experiments were carried out three times. Supplementary Figure S3. Migration (A) and Invasion (B) of MDA-MB-231 cells transfected with shCtrl or shFZD7 was analyzed by Wound healing and Transwell, respectively. (C) Invasion of Hs578T cells transfected with shCtrl or shFZD7 was analyzed by Transwell. (D) Migration of MCF7 cells transfected with control vector or FZD7 overexpression vector was analyzed by Wound healing. All experiments were carried out three times. Supplementary Figure S4. (A) Interrogation of CCLE database showed the correlation of FZD7 with CD44, LGR5, EGFR and NOTCH2 in BC cell lines. (B) Interrogation of GSE12777 database showed the correlation of FZD7 with CD44, EGFR and NOTCH2 in BC cell lines. (C) Interrogation of GSE2603 database showed the correlation of FZD7 with LGR5, EGFR and NOTCH2 in BC tissues. Supplementary Figure S5. (A) Mammosphere formation in Hs578T cells transfected with shCtrl or shFZD7 was shown. (B) The fraction of Lgr5+ subpopulation in Hs578T cells transfected with shCtrl or shFZD7 was determined by flowcytometry. (C) Expression of CD [file 12964_2020_646_MOESM2_ESM.docx]

**Supplementary Figure S1**

**
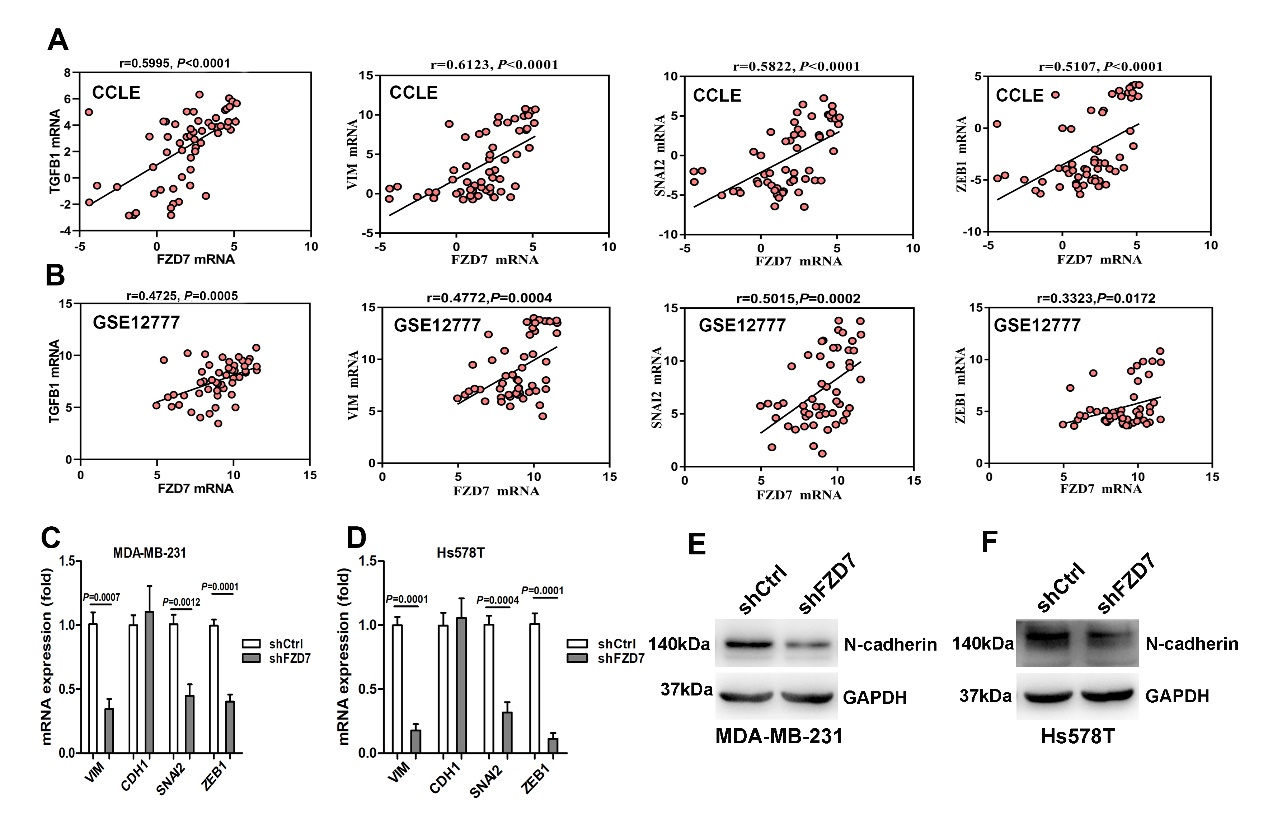
Supplementary Figure S1.** Interrogation of CCLE database (A) and GSE12777 database (B) showed the correlation of FZD7 with TGFB1, VIM, SNAI2 (Slug) and ZEB1 in BC cell lines. Expression of VIM, CDH1, SNAI2 and ZEB1 was detected in MDA-MB-231 (C) and Hs578T (D) cells transfected with shCtrl or shFZD7 by real-time PCR. Expression of N-cadherin was detected in MDA-MB-231 (E) and Hs578T (F) cells transfected with shCtrl or shFZD7 by Western blot. All experiments were carried out three times. Data are expressed as Mean±s.e.m.

**Supplementary Figure S2**

**
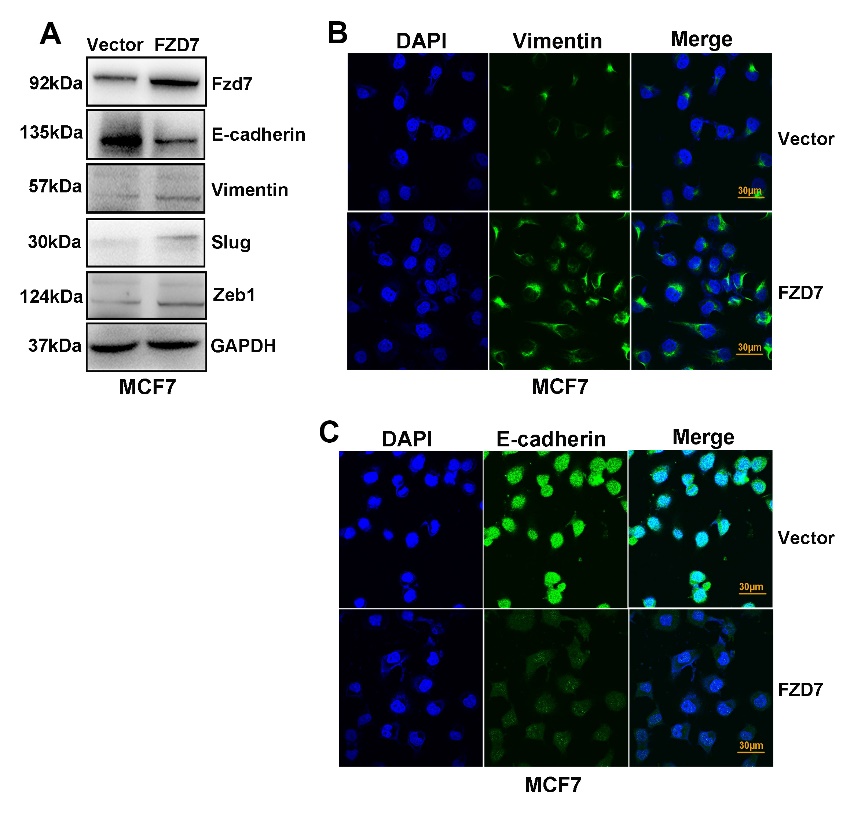
**

**Supplementary Figure S2.** (A) Expression of Fzd7, Vimentin, E-cadherin, Slug and Zeb1 was detected in MCF7 cells transfected with control vector or FZD7 overexpression vector by Western blot. Expression of Vimentin (B) and E-cadherin (C) was detected in MCF7 cells transfected with control vector or FZD7 overexpression vector by Immunofluorescence staining. All experiments were carried out three times.

**Supplementary Figure S3**

**
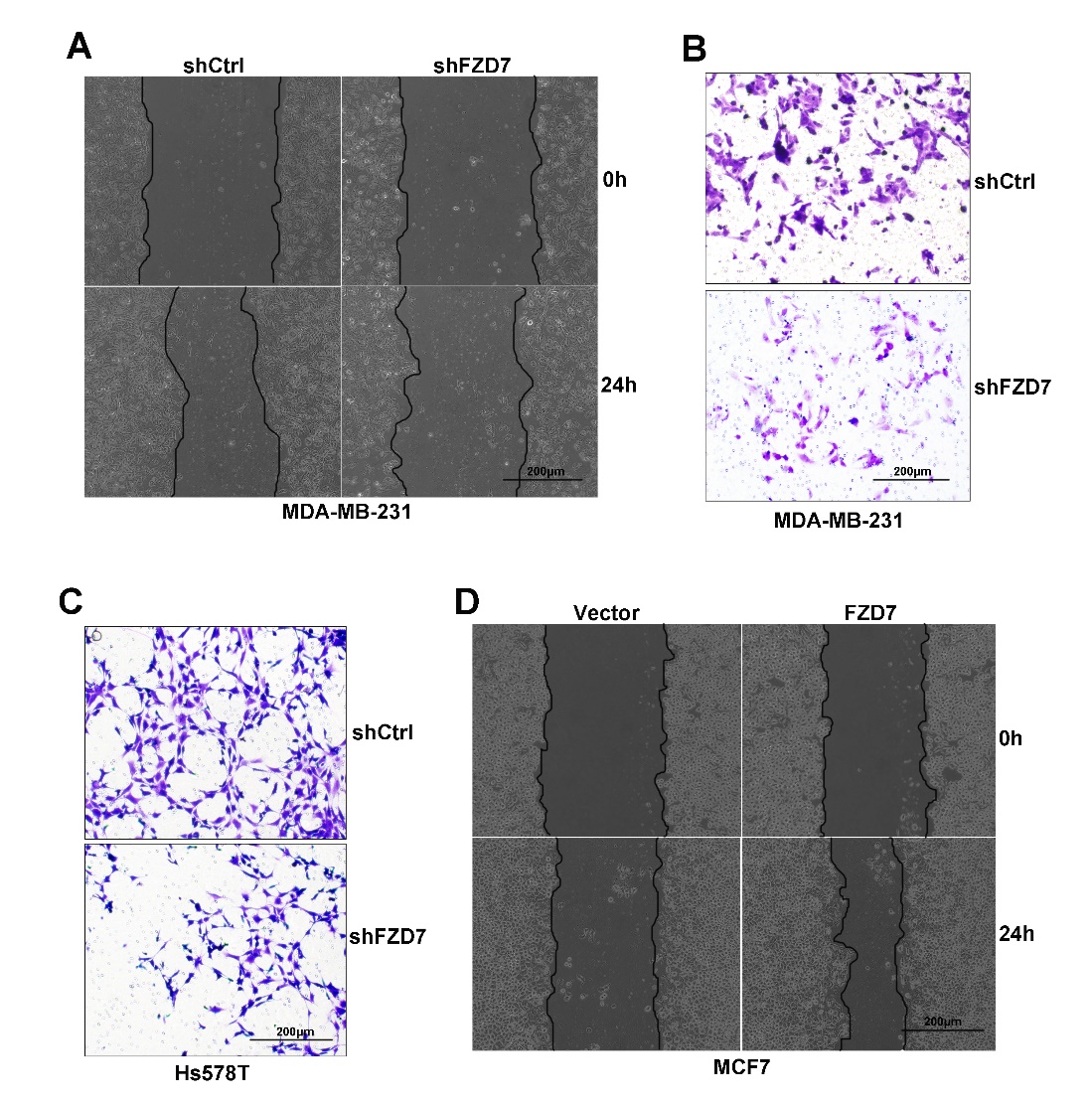
Supplementary Figure S3.** Migration (A) and Invasion (B) of MDA-MB-231 cells transfected with shCtrl or shFZD7 was analyzed by Wound healing and Transwell, respectively. (C) Invasion of Hs578T cells transfected with shCtrl or shFZD7 was analyzed by Transwell. (D) Migration of MCF7 cells transfected with control vector or FZD7 overexpression vector was analyzed by Wound healing. All experiments were carried out three times.

**Supplementary Figure S4**

**
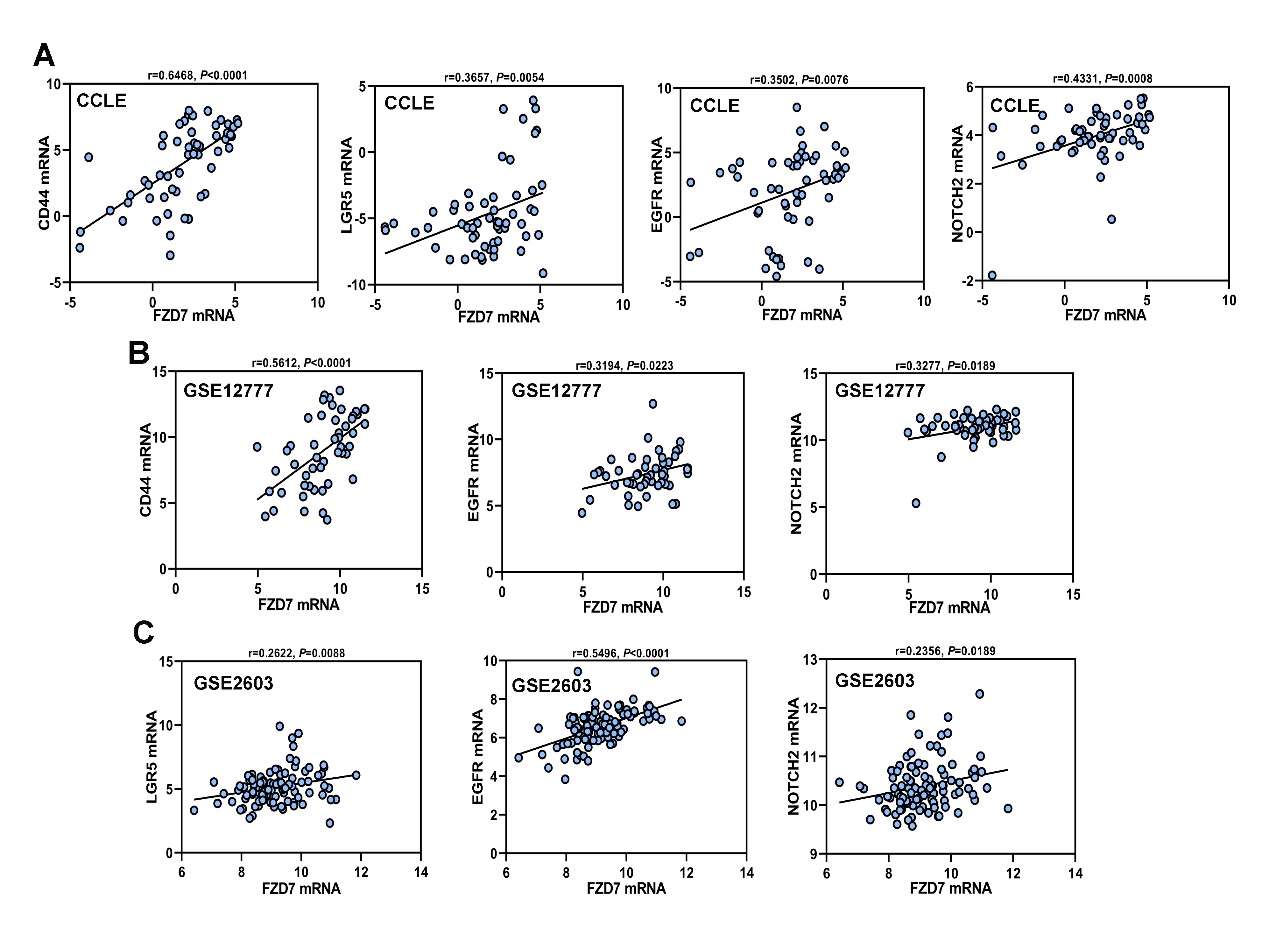
Supplementary Figure S4.** (A) Interrogation of CCLE database showed the correlation of FZD7 with CD44, LGR5, EGFR and NOTCH2 in BC cell lines. (B) Interrogation of GSE12777 database showed the correlation of FZD7 with CD44, EGFR and NOTCH2 in BC cell lines. (C) Interrogation of GSE2603 database showed the correlation of FZD7 with LGR5, EGFR and NOTCH2 in BC tissues.

**Supplementary Figure S5**

**
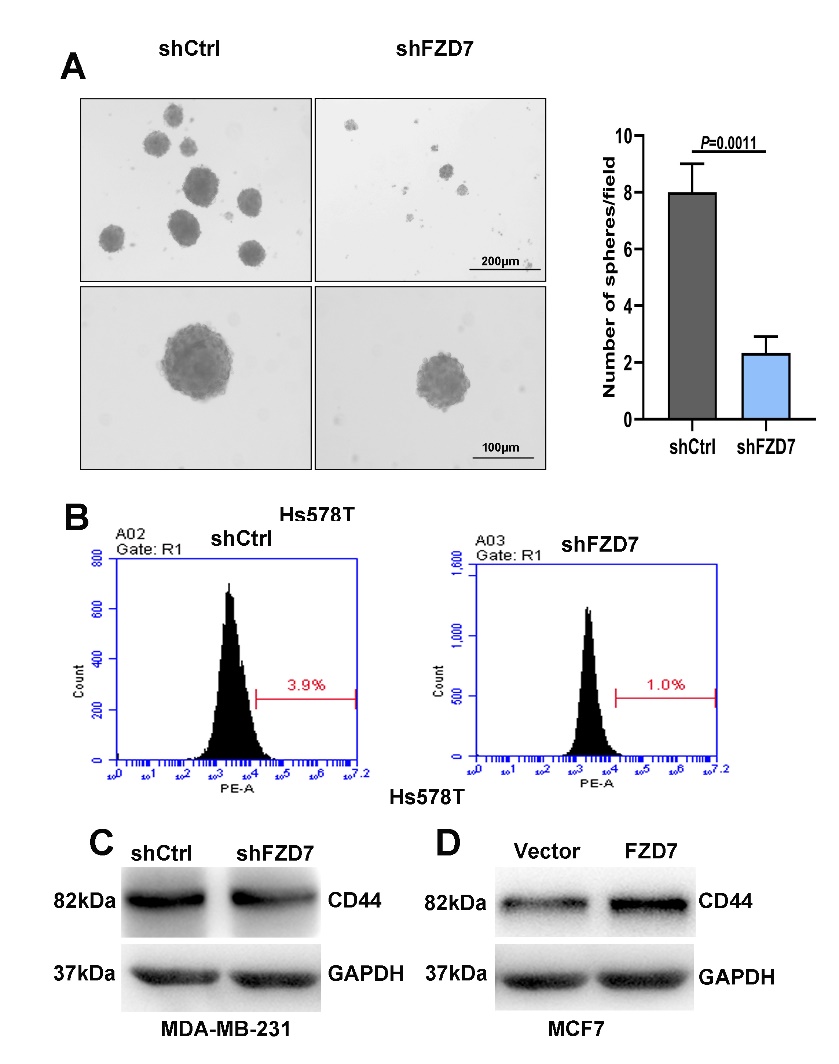
**

**Supplementary Figure S5.** (A) Mammosphere formation in Hs578T cells transfected with shCtrl or shFZD7 was shown. (B) The fraction of Lgr5^+^ subpopulation in Hs578T cells transfected with shCtrl or shFZD7 was determined by flowcytometry. (C) Expression of CD44 was detected in MDA-MB-231 cells transfected with shCtrl or shFZD7 by Western blot. (D) Expression of CD44 was detected in MCF7 cells transfected with control vector or FZD7 overexpression vector by Western blot. All experiments were carried out three times. Data are expressed as Mean±s.e.m.

**Supplementary Figure S6**

**
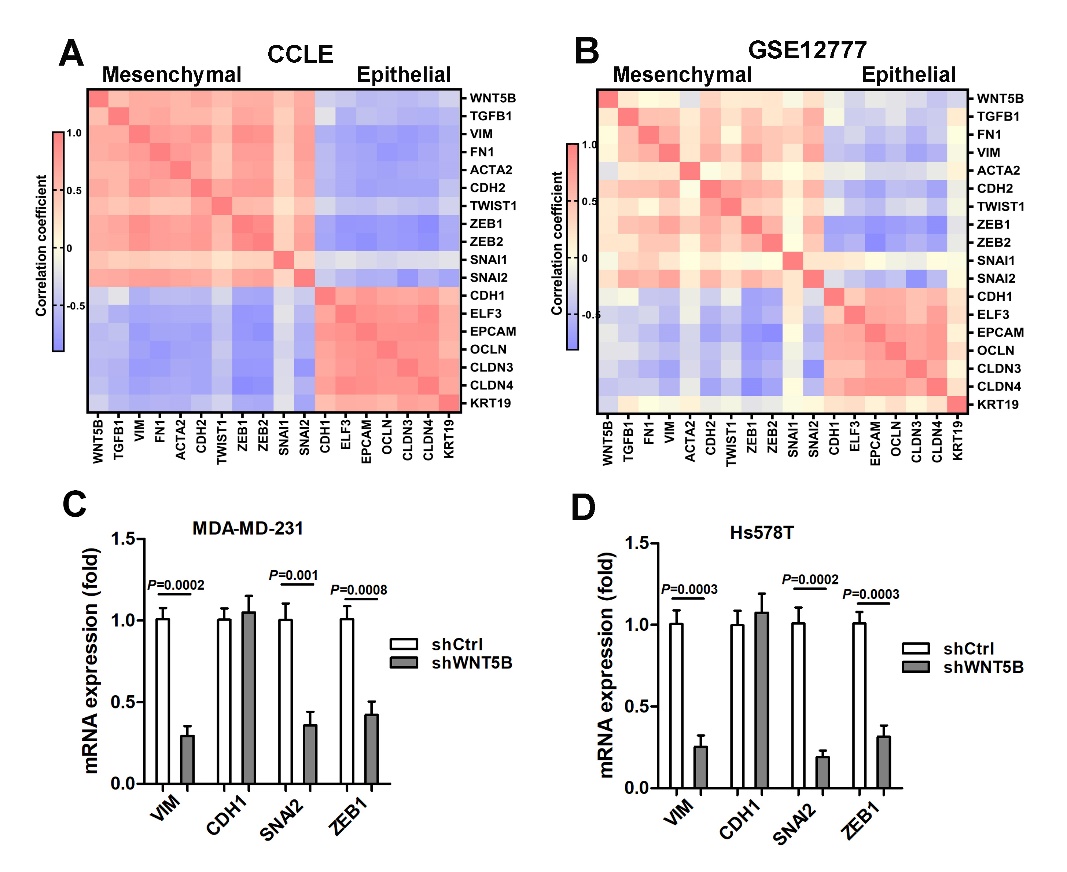
Supplementary Figure S6.** Heat maps generated from CCLE database (A) and GSE12777 database (B) demonstrated the correlation of WNT5B with mesenchymal-related genes and epithelial-related genes in human BC cell lines. Expression of VIM, CDH1, SNAI2 and ZEB1 was detected in MDA-MB-231 (C) and Hs578T (D) cells transfected with shCtrl or shWNT5B by real-time PCR. All experiments were carried out three times. Data are expressed as Mean±s.e.m.

**Supplementary Figure S7**

**
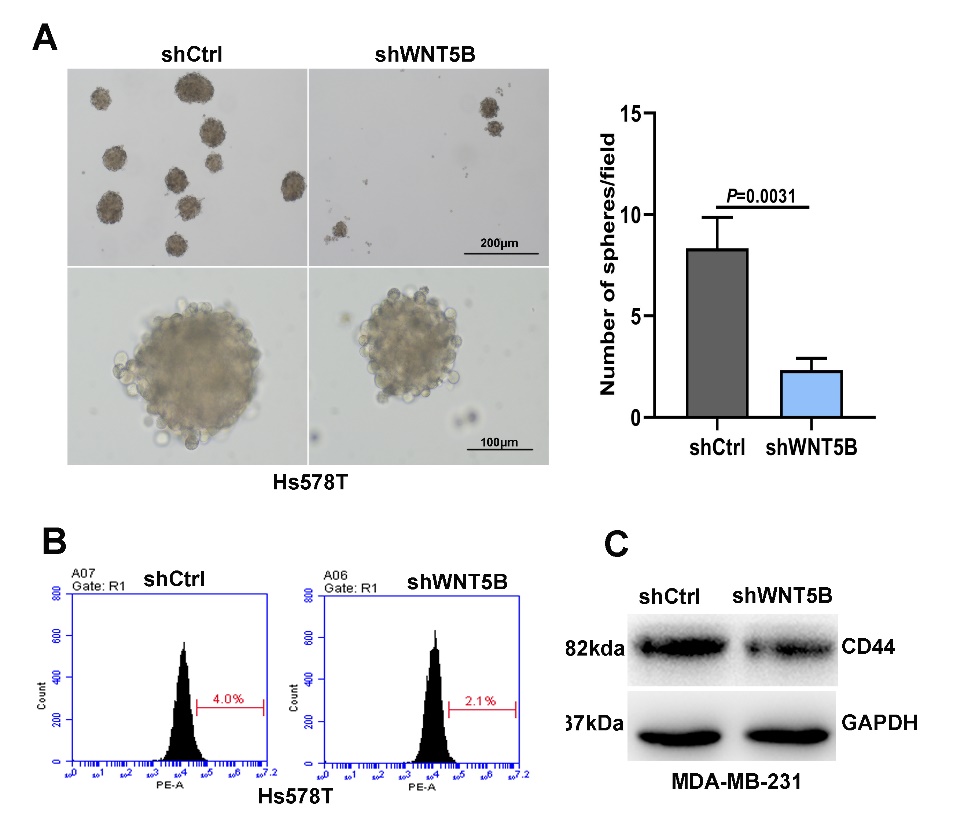
**

**Supplementary Figure S7.** (A) Mammosphere formation in Hs578T cells transfected with shCtrl or shWNT5B was shown. (B) The fraction of Lgr5^+^ subpopulation in Hs578T cells transfected with shCtrl or shWNT5B was determined by flowcytometry. (C) Expression of CD44 was detected in MDA-MB-231 cells transfected with shCtrl or shWNT5B by Western blot. All experiments were carried out three times. Data are expressed as Mean±s.e.m.

**Supplementary Figure S8**

**
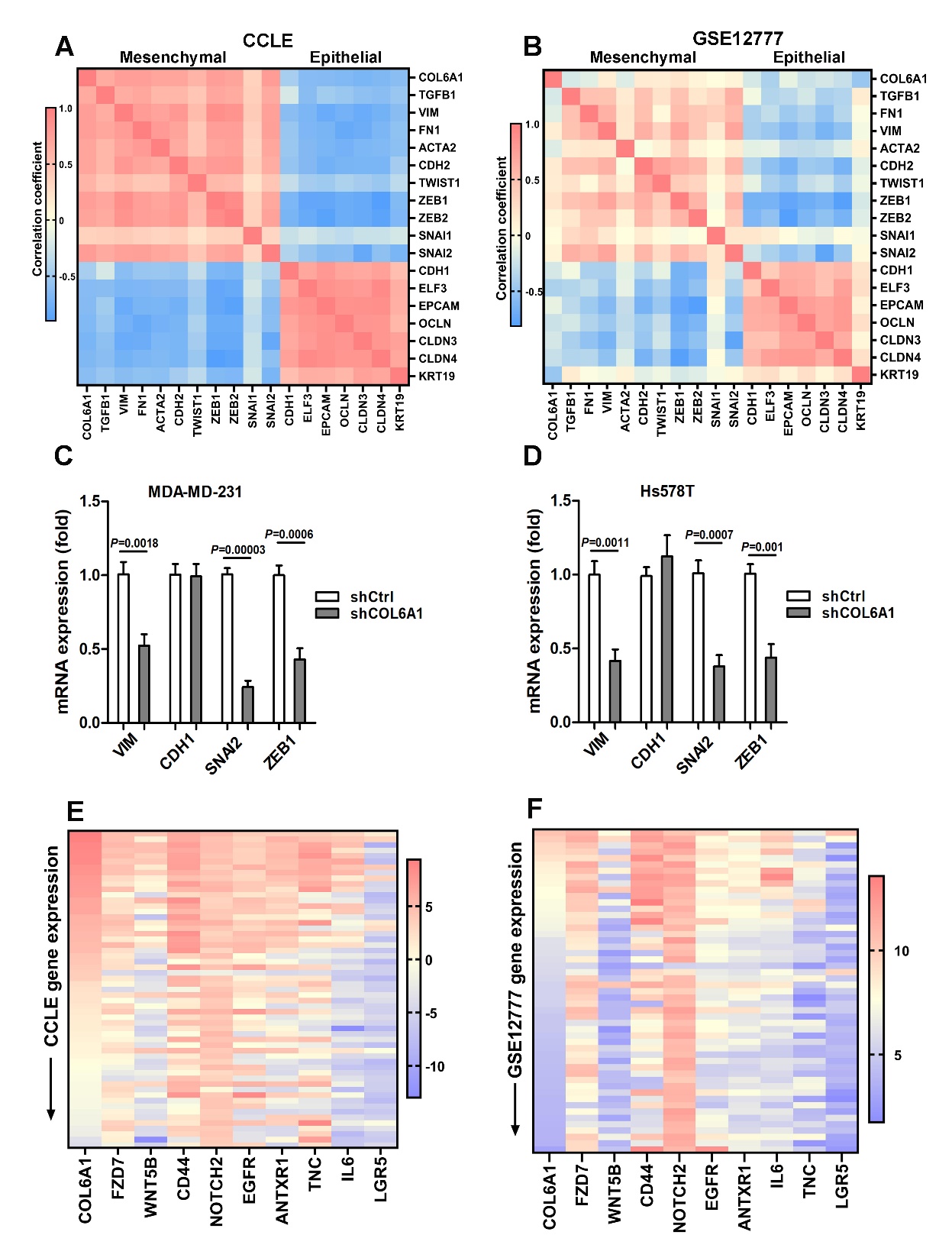
Supplementary Figure S8.** Heat maps generated from CCLE database (A) and GSE12777 database (B) demonstrated the correlation of COL6A1 with mesenchymal-related genes and epithelial-related genes in human BC cell lines. Expression of VIM, CDH1, SNAI2 and ZEB1 was detected in MDA-MB-231 (C) and Hs578T (D) cells transfected with shCtrl or shCOL6A1 by real-time PCR. Heat maps generated from CCLE database (E) and GSE12777 database (F) demonstrated the correlation of COL6A1 with stemness-related genes in human BC cell lines. All experiments were carried out three times. Data are expressed as Mean±s.e.m.
